# Syntactically-informed word representations from graph neural network

**DOI:** 10.1016/j.neucom.2020.06.070

**Published:** 2020-11-06

**Authors:** Thy Thy Tran, Makoto Miwa, Sophia Ananiadou

**Affiliations:** a National Centre for Text Mining, Department of Computer Science, The University of Manchester, Manchester, United Kingdom; b Toyota Technological Institute, Nagoya, Japan; c Artificial Intelligence Research Centre, National Institute of Advanced Industrial Science and Technology (AIST), Tokyo, Japan; d The Alan Turing Institute, London, United Kingdom

**Keywords:** Natural language processing, Contextual word representation, Word representation, Word embedding, Syntactic word representation

## Abstract

Most deep language understanding models depend only on word representations, which are mainly based on language modelling derived from a large amount of raw text. These models encode distributional knowledge without considering syntactic structural information, although several studies have shown benefits of including such information. Therefore, we propose new syntactically-informed word representations (SIWRs), which allow us to enrich the pre-trained word representations with syntactic information without training language models from scratch. To obtain SIWRs, a graph-based neural model is built on top of either static or contextualised word representations such as GloVe, ELMo and BERT. The model is first pre-trained with only a relatively modest amount of task-independent data that are automatically annotated using existing syntactic tools. SIWRs are then obtained by applying the model to downstream task data and extracting the intermediate word representations. We finally replace word representations in downstream models with SIWRs for applications. We evaluate SIWRs on three information extraction tasks, namely nested named entity recognition (NER), binary and *n*-ary relation extractions (REs). The results demonstrate that our SIWRs yield performance gains over the base representations in these NLP tasks with 3–9% relative error reduction. Our SIWRs also perform better than fine-tuning BERT in binary RE. We also conduct extensive experiments to analyse the proposed method.

## Highlights

• Syntactically-informed representations based on static and/or contextual representations.
• Pre-encode syntactic information with automatically annotated data.
• Improve performance over base representations on three information extraction tasks.
• Syntactic dependencies can be beneficial for both static and contextual embeddings.
• Easily adapt in different linguistic tasks than fine-tuning large models.

## 1 Introduction

Word representations have been widely used in natural language processing (NLP) tasks. Most approaches rely on language models (LMs) to obtain static word representations [1], [2], [3], which conflate all possible meanings of a word in a single real-valued vector. Recent work investigated contextualised word representations, which assign a different representation to each occurrence of a word based on its local context [4], [5]. These contextual word representations have demonstrated improvements in downstream tasks over the static ones. Alternatively, large-scale LMs have been proposed to use in downstream application models with fine-tuning approaches [6], [7], [8]. These fine-tuning methods have shown promising results with higher performance than contextual word representations in some applications such as text classification and textual entailment [7]. However, not all tasks can be easily represented by large-scale LMs, therefore required an additional model architecture to be designed on top of them [9], [10]. On the contrary, contextual representations can be easily adopted as plug-in plug-out features. Also, contextual representations are cheaper to run as they are pre-computed once only for each instance and run in many experiments with smaller models on top. In other words, the computational costs for fine-tuning methods are much higher than contextual approaches.

**Fig. 1:**
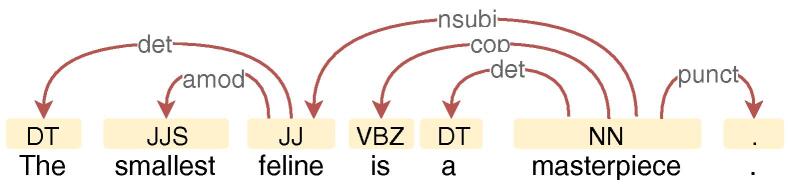
A sentence with its part-of-speech tags (yellow rectangles) and syntactic dependency annotations (red connections).

All of the LMs mentioned above are mainly trained on a large amount of raw text, and thus do not explicitly encode any linguistic structures. Recent studies have shown that downstream task performance may benefit from linguistic structures such as syntactic information [11], [12], even when contextual word representations and pre-trained models are also used [13], [14], [15]. The syntactic information, i.e., part-of-speech (POS) tags and dependencies (see example in Fig. 1; in this paper, we use the term “syntactic information” interchangeably with “POS tags” and “dependencies”), has been well studied and can be obtained efficiently with high accuracy using existing dependency parsing tools [16], [17]. Many task-oriented neural models do not take into account such syntactic information despite potential performance gains. To include such information to existing models, it is necessary to change the model architecture. This leads to the following research question: *Is there a universal way to include syntactic bias into the model without changing the architecture while retaining large-scale information?* In this paper, we will demonstrate that syntax can be pre-encoded with contextual word representations which can be beneficial for subsequent applications.

We introduce **S**yntactically-**I**nformed **W**ord **R**epresentations (**SIWRs**) that can incorporate syntactic information in neural models without explicitly changing their architecture. Syntactic information is integrated into existing word representations such as GloVe [18], ELMo [5] and BERT [19] by learning from automatically annotated data which are task-independent. We propose **the SIWR model** extends a graph convolutional neural network (GCN) and builds on top of these word representations. Since in English word order is important, we preserve it by adding these connections into the graph layer. We then obtain SIWRs from the pre-trained SIWR model using a contextualised word representation extraction scheme. Finally, we incorporate SIWRs into downstream models by only replacing the word representations with SIWRs. We show that SIWRs enrich the base word representations with syntactic information and boost the performance in downstream tasks. Unlike previous work [20], our findings demonstrate that syntactic information is helpful in the advent of contextual word representations and large pre-trained models.

**Fig. 2:**
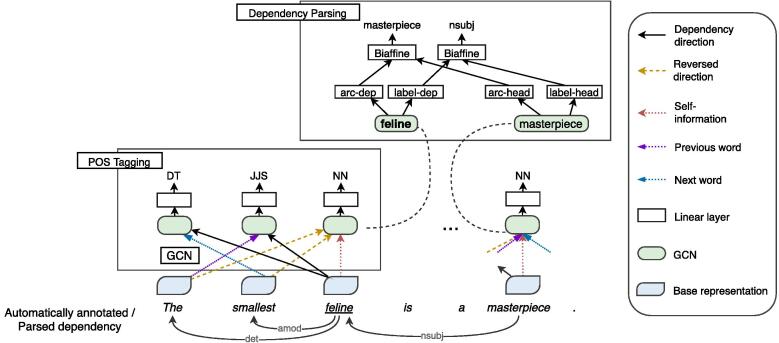
Overview of our neural graph model. The model addresses syntactic tasks to incorporate syntactic information into word representations. We only show one graph convolution neural layer for simplicity.

Fig. 2 shows the architecture of our SIWR model. We first prepare pre-trained static and contextual word representations, e.g., GloVe, ELMo, and contextual BERT, as the base representations. We then feed them to our SIWR model, which consists of a two-stacked GCN layer over dependency trees along with self and sequential information. The GCN is used to include syntactic information into the base word representations. The SIWR model jointly predicts part-of-speech (POS) tags and syntactic dependencies. We only pre-train the SIWR model once with a relatively modest amount of task-agnostic data that are automatically annotated by using existing syntactic tools. Once the SIWR model is obtained, we apply the model to downstream task data and obtain SIWRs by combining the outputs of all layers in the model. We simply replace word representations in downstream task models with SIWRs to cater for different applications.

We compare the enriched SIWRs with their base representations ELMo [5] and biomedical word embeddings (PubMed) [21] on the existing models in three downstream NLP tasks: (a) nested named entity recognition (nested NER), (b) relation extraction (RE) and (c) *n*-ary relation extraction (*n*-ary RE). SIWRs show improvements over the base representations achieving the following relative error reductions: 3.79% in F1-score for nested NER, 6.64% in F1-score for RE, and 6.98% of accuracy for *n*-ary RE, which results in comparable performance to the state-of-the-art on the three tasks. In addition, we implement BERT [19] in both contextual and fine-tuning methods on nested NER and binary RE. We also employ BERT as the base representations in our SIWRs_BERT_ for comparison. Surprisingly, our SIWRs_BERT_ based on contextual BERT even perform better than the fine-tuning in binary RE with the F1-score of 72.45% and 66.84% respectively. Meanwhile, our enhanced representations perform comparably to the fine-tuning BERT with less training parameters in the nested NER with the F1-score of 82.06% and 82.84% respectively. Our extensive analysis also shows that the syntactic inductive bias can be easily transferred to subsequent NLP tasks and beneficial for performance improvement.

The contributions of our work are as follows:

• We propose a method for the construction of syntactically-informed representations (SIWRs) based on static and/or contextual representations.
• SIWRs allow us to incorporate syntactic information into existing NLP neural models simply by replacing the original word representations, without altering the architecture of these models with a relatively modest amount of syntactically annotated data.
• We demonstrate that SIWRs improve the performance over base representations on three downstream NLP tasks: nested NER, binary RE, and *n*-ary RE. The improvement can be obtained with only a small number of weight parameters compared with training task-oriented syntactic representations in downstream models.
• Extensive analysis of SIWRs over base representations indicates that syntactic dependencies can be beneficial for both static and contextual embeddings, contrary to previous findings [20].
• Last, we show that our enhanced representations can be transferred more easily to different linguistic tasks than fine-tuning large-scale language models in downstream tasks that require inference.

## 2 SIWRs: syntactically-informed word representations

We propose the SIWR model that utilises GCN as its core component to integrate syntactic information into word representations as illustrated in Fig. 2. The syntactic information includes part-of-speech (POS) tags and dependency connections. Once the SIWR model is trained, we apply it to downstream task data and extract SIWRs from the representations of all layers. The resulting word representations can be used to improve downstream NLP models similar to contextualised word representations.

We first describe the base representations used in our model which can be either static or contextualised word representations. We then introduce the SIWR model, following by a description for pre-training the SIWR model. At last, we present how SIWRs be extracted and used in downstream NLP models.

### 2.1 Base Representations

As shown in Fig. 2, pre-trained word representations are used as input of our model, namely base representations. Base representations can be either static or contextualised word representations. Static word representations represent a word by a single continuous vector regardless of the context where the word occurs. If base representations are pre-trained static representations, we create an embedding layer that maps each word to a single continuous vector $e_{s}\in R^{d_{w}}$, where $e_{s}$ is the static embedding and $d_{w}$ is the dimension of the word embedding. In contrast, in contextualised word representations, word vectors change dynamically based on context. To use contextualised word representations, we construct the base representations $e_{c}\in R^{d_{w}}$ by combining intermediate word-based vectors ($L\times e_{i}\in R^{d_{w}}$) from a pre-trained language model:

(1) $$e_{c}=\overset{L}{\underset{i=0}{\sum }}\gamma_{i}e_{i},$$

where γ are softmax-normalised weights of *L* layers, the vector $e_{i}$ is the internal word embedding from the *i*th layer in the pre-trained model. These base representations are obtained from existing pre-trained LMs, which are fixed during pre-training and then are transferred in downstream tasks.

### 2.2 Part-of-speech tags and dependencies

We consider two types of syntactic information: part-of-speech tags and dependencies (see Fig. 1) which are two fundamental syntactic features and a prerequisite for further NLP analysis. POS tags are necessary for identifying the essential components of a sentence e.g., nouns and verbs. Dependencies provide information on relations between words in a sentence, which can be leveraged to extract the subject, object, and their modifiers in a sentence.

### 2.3 The SIWR model

The goal of our SIWR model is to incorporate dependency context into word representations. To achieve this, we utilise the dependency tree structure of a sentence, where nodes correspond to words and edges are dependency relations between words. We also preserve the word order by adding *previous* and *next* word connections to the word graph. GCN has been successfully used for operating on labeled word graph in previous work [22], [23], we hence follow to use the edge-wise gating GCN. We create five edge types (Fig. 2): *dependent* edges for head-to-dependent connections (*dependency direction*), *reversed dependent* edges for dependent-to-head direction (*reversed direction*), *previous* and *next* word connections, as well as *self-informed* edges to retain information from the word. The representation $h_{v}^{k+1}$ of word *v* at layer k+1 is defined as follows:

(2) $$h_{v}^{k+1}=\text{ReLU}\left(\underset{u\in N\left(v\right)}{\sum }g_{u,v}^{k+1}\left(h_{u}^{k}W_{edge\left(u,v\right)}^{k}+b_{\mathrm{dep}\left(u,v\right)}^{k}\right)\right),$$

where N(v) denotes the set of words that directly connect to word $v,h_{v}^{*}\in {\mathbb{R}}^{d_{w}}$ is the representation of word $v\text{;}W_{edge(u,v)}^{*}\in {\mathbb{R}}^{d_{w}\times d_{w}}$ is a weight matrix that learned the connective information from word *u* to word *v*; and $b_{dep(u,v)}^{*}\in {\mathbb{R}}^{d_{w}}$ is the dependency type embedding of the edge (u,v) if edge (u,v) is a dependent or reversed dependent edge, otherwise ${\overset{}{b}}_{dep(u,v)}^{*}$ is the edge type embedding. The gate $g_{u,v}$ controls the information weight of edge (*u,v*), which is calculated as follows:

(3) gu,vk+1=σhukW^edgeu,vk+b^depu,vk,

where W^egde(u,v)*∈ℝdw and b^dep(u,v)*∈ℝ1 function similarly as weight ${\overset{}{W}}_{egde(u,v)}^{*}$ and bias ${\overset{}{b}}_{dep(u,v)}^{*}$ in Eqn 2 and σ is the sigmoid function. Since GCN only encodes information into a word from its adjacent nodes in the word graph, we employ two-stacked GCN layers to capture longer dependency context [22], [23].

### 2.4 Pre-training the SIWR model

We pre-train our SIWR model to jointly capture POS tags and syntactic dependencies . In particular, for each word we predict its *POS tag* and and its dependency connection, i.e., *head* of the word (*dependent arc*) and the *label* of the dependent arc. We first construct POS representation $h_{v}^{\mathrm{POS}}$ of word *v* for POS prediction. We then construct arc and label representations of word *v* as a dependent, $h_{v}^{ar\mathrm{c\_de}p}$ and $h_{v}^{\mathrm{head}\mathrm{\_de}p}$, respectively. Similarly, we compute arc and label representations of word *v* when serving as a head word, $h_{v}^{ar\mathrm{c\_de}p}$ and $h_{v}^{\mathrm{label}\mathrm{\_head}}$, respectively. For simplicity, we denote these representations as $h_{v}^{\mathrm{syn}}$, and compute them as follows.

(4) $$h_{v}^{\mathrm{syn}}=\text{f}\left(h_{v}^{L}W^{\mathrm{syn}}+b^{\mathrm{syn}}\right),$$

where $h_{v}^{L}$ is the output of the last GCN layer corresponding to a word *v* and syn∈{POS, arc_head, arc_dependent, label_head, label_dependent}. f is an activation function, which is tanh for POS representations and ReLU for dependency representations.

The POS representation $h_{v}^{\mathrm{POS}}$ of a word *v* is then fed into a softmax classifier to predict the correct POS tag of the target word. We optimise the negative log-likelihood objective of POS tagging (${\text{J}}_{\mathrm{POS}}$) as follows:

(5) $${\text{J}}_{\mathrm{POS}}=-\underset{v}{\sum }\log p\left(y_{v}^{\mathrm{POS}}\right),$$

where $p\left(y_{v}^{\mathrm{POS}}\right)$ is the probability value that the correct POS label is assigned to a word *v*.

Meanwhile, to predict syntactic dependencies, we need to predict (i) heads of words (dependency arcs) and (ii) arc labels. First, we predict the head of word *v* (dependent arc) $s_{v}^{\mathrm{arc}}$ by passing the arc_dep representation $H^{\mathrm{arc}\_\mathrm{dep}}$ of word *v* and arc_head representations $H^{\mathrm{arc}\_\mathrm{head}}$ of other words in a sentence to a biaffine classifier [24] (Eqn 6). Likewise, we use another biaffine layer for label prediction (Eqn 7). In particular, we pass the label_dep representation $h_{v}^{\mathrm{label\_dep}}$ of word *v* and label_head representations $H^{\mathrm{label}\_\mathrm{head}}$ of other words to the biaffine classifier, resulting in the label probability $s_{v}^{{\mathrm{label}}_{i}}$. The label probability is then multiplied by the predicted arc probability $s_{v}^{\mathrm{arc}}$ to get the final arc-label probability $s_{v}^{{\mathrm{arc\_label}}_{i}}$ (Eqn 8). The computation is as follows:

(6) $$s_{v}^{\mathrm{arc}}=H^{arc\_\mathrm{he}ad}W^{\mathrm{arc}}h_{v}^{arc\_\mathrm{de}p}$$

(7) $$s_{v}^{{\mathrm{label}}_{i}}=H^{\mathrm{label}\_\mathrm{head}}W^{{\mathrm{label}}_{i}}h_{v}^{\mathrm{label\_dep}}$$

(8) $$s_{v}^{{\mathrm{arc\_label}}_{i}}=s_{v}^{{\mathrm{label}}_{i}}⊙s_{v}^{\mathrm{arc}},$$

where $H^{*}=\{h_{1}^{*},\ldots ,h_{n_{s}}^{*}\}\in {\mathbb{R}}^{n_{s}\times d_{w}}\;\text{is the matrix whose rows are arc\_head representations from Eqn 4},W^{*}\in {\mathbb{R}}^{d_{w}\times d_{w}}$, and $h_{v}^{*}\in {\mathbb{R}}^{d_{w}}$, which results in $s^{\mathrm{arc}},s^{{\mathrm{label}}_{*}}\in R^{n_{s}}$ with $n_{s}$ is the number of words in a sentence and ⊙ is element-wise multiplication. We design the model that performs similarly to an auto-encoder (taking the dependency graph as input and predicting it later) to ensure that dependency information is embedded in the representations. We also employ the negative log-likelihood to train dependency parsing (${\text{J}}_{\mathrm{DP}}$), the objective function is defined as follows:

(9) $${\text{J}}_{\mathrm{DP}}=-\underset{v}{\sum }\log p\left(y_{v}^{\mathrm{arc\_label}}\right),$$

where $p\left(y_{v}^{\mathrm{arc\_label}}\right)$ is the probability assigned the correct head word and the correct dependency label to word *v*. We jointly train the model by maximising both POS tagging and dependency parsing, i.e., minimising the following loss,

(10) $$\text{J}={\text{J}}_{\mathrm{POS}}+{\text{J}}_{\mathrm{DP}}+\lambda ||W|{|}^{2},$$

where $\lambda ||W|{|}^{2}$ is the L2-norm regularisation term, and λ is a hyperparameter and W includes all learnable weights in our model.

### 2.5 SIWRs Extraction and application

Within a specific domain, we only need to train the SIWR model once, to compute word representations for downstream linguistic tasks. SIWRs are modelled as a weighted combination of the intermediate embeddings: the base representations and the outputs of the two-stacked graph convolutional neural layers in the SIWR model. The base representations provide information derived from large-scale data, while the other layers include syntactic information and long-range dependency context. We employ the combination scheme of contextualised representations in Eq. (1),

(11) $$\mathrm{SIWRs}=\overset{L+2}{\underset{i=0}{\sum }}\beta_{i}e_{i},$$

where *L* is equal to 1, if the base representations are static embeddings, or *L* is the number of intermediate layers if the base representations are contextualised embeddings, and +2 from two-stacked GCNs. $\beta \in R^{L+2}$ functions as γ in Eq. (1), which is trained in downstream tasks while the intermediate embeddings serve as the input and are fixed during training.

## 3 Evaluation benchmark

We evaluated SIWRs on three downstream tasks: nested named entity recognition (nested NER), binary relation extraction (RE) and *n*-ary relation extraction (*n*-ary RE). The reason we opted for these tasks is that in current neural NER and RE methods, syntactic information is omitted. In the case of N-ary RE, the state-of-the-art (SOTA) model [29] includes syntactic information, we removed the syntactically related part of the SOTA and replaced the word representations by our SIWRs for comparison. This demonstrates the comparison of the syntactic information in our SIWRs with directly incorporating syntactic in a task-oriented model. We provide in Table 1, a summary of the data and evaluation settings e.g., evaluation tasks, data sets, baselines, and state-of-the-art models.

**Table 1:** Evaluation benchmark, our implementation baselines as well as current state-of-the-art models.

Task	Dataset	Annotation	Baseline	SOTA
Nested NER	ACE 2005	Entity	Layered [25]	Merge and Label [26]
Binary RE	ACE 2005	Binary relation	Walk [27]	BIO tag [28]
*N*-ary RE	Drug-Gene-Mut.	Ternary relation	BiLSTM [29]	Graph LSTM [29]

We used a general domain dataset, ACE2005 [30] for nested NER and binary RE. For *n*-ary RE, we used the biomedical drug-gene-mutation dataset in [29] since there is no *n*-ary RE dataset in general domain. We used evaluation metrics include Precision (P), Recall (R) and F1-score (F1) as previous work to evaluate both nested NER and binary RE tasks. Regarding *n*-ary RE, we followed previous work to use accuracy (%) as an evaluation metric. In the following sections, we briefly explain the evaluation tasks, the data, the baselines we used and related work corresponding to each task.

### 3.1 Nested named entity recognition

Nested named entity recognition (nested NER) detects complex entities that include both flat and nested entities, i.e., embedded entities included in other entities. We evaluated the performance of nested NER on the ACE2005 dataset annotated with 7 nested entity types: person (PER), location (LOC), organisation (ORG), Geo-PoliticalEntity (GPE), facility (FAC), vehicle (VEH) and weapon (WEA). We follow Ju et al. [25] in data splitting by keeping the 8:1:1 ratio for training, development and testing datasets, respectively. We also used the conventional BIO tagging scheme in this experiment.

The stacked bidirectional long short-term memory - conditional random field (BiLSTM-CRF) model proposed in Ju et al. [25] was used as our baseline since it achieves top performance without using syntactic information. It is also publicly available.^1^ The model extracts nested entities by dynamically stacking flat BiLSTM-CRF blocks to predict entities from inside out. This allows the model to encode the dependencies between nested entities.

We also compared our results with the state-of-the-art model in Fisher and Vlachos [26]. The model predicts real-valued segmentation structures for nested NER using a merge and label approach. The method includes two stages: firstly, it detects the entity boundaries at all nested levels; secondly, the embeddings of predicted entities are then used to predict their entity labels.

### 3.2 Relation extraction

Relation extraction (RE) identifies relations between two entity mentions in a sentence. We evaluated our representations on the ACE2005 dataset that provides a total of 6 relation types: *ORG-AFF*, *PHYS*, *PART-WHOLE*, *GEN-AFF*, *ART* and *PER-SOC*; and a specific *NA* category to indicate no relation. During testing, we only evaluated 6 relation types.

For this task, we employed the walk-based model proposed in Christopoulou et al. [27] as our baseline.^2^ The walk model supports relation detection by leveraging interactions between all entities in a sentence. In particular, entities are formed as nodes in a graph structure where edges are considered as relation paths. Then, a relation candidate embedding is constructed by walking from the head entity to the tail entity through different path lengths. The constructed embeddings are then fed into a softmax classifier to identify the underlying relation.

We compared our baselines using different base representations and corresponding SIWRs to related work [11], [27], [28]. Miwa and Bansal [11] integrated dependencies in a long short-term memory (LSTM) model, while we used the model proposed by Christopoulou et al. [27] as our baseline. For reference, we also showed the results of Ye et al. [28] on the ACE2005 dataset. They investigated the BIO tags from named entity recognition which may give a hint to the relation extraction. However, they employed a slightly different evaluation setting compared to ours, the details will be explained in the fifth paragraph in §5.

### 3.3 *N*-ary relation extraction

*N*-ary relation extraction (*n*-ary RE) is fundamentally an extension of the RE task, which further detects relations among several entity mentions in a sentence. For this task, we used the drug-gene-mutation data set [29] with the same split as the released data.^3^ We followed [29] to evaluate the task using fivefold cross-validation where 200 instances from the training set were randomly selected for development in each fold.

We adopted the LSTM model in [29] as the baseline for our experiments. The LSTM model does not include syntactic information. We replaced its word representations with our pre-encoded syntactic word representations to compare with their proposed graph-based LSTM that included such information during downstream task training.

We did not compare with Song et al. [31] since we found that they did not blind entity names in their experiments. When we replaced entities by their entity types, Song’s model performance was lower than Peng et al. [29], 69.36%, and 77.9% respectively. Therefore, we opted to use the graph-based LSTM result in Peng et al. [29] as the previous SOTA.

## 4 Experimental settings

We performed nested NER, binary RE, and *n*-ary RE using different word representations including static (GloVe and PubMed), contextual embeddings (ELMo and BERT) and our SIWRs_{PubMed, ELMo, BERT}_. In the following, we demonstrate the training settings of our SIWR model, the base representations, and training data for each task as well as the experimental settings.

### 4.1 The SIWR model training settings

We implemented our SIWR model using the Theano library [32] trained using Adam optimiser with default hyperparameters [33]. During pre-training, we implemented gradient clipping, dropout, and L2 regularisation to avoid over-fitting. We also incorporated early stopping with patience equal to 10 updating steps. We froze the base representations over all pre-training processes. The hyperparameters which were tuned for our model are listed in Table 2. We tuned them by applying the Tree Parzen Estimator optimization algorithm using the Hyperopt toolkit.^4^

**Table 2:** Value range and best value of tuned hyperparameters. Dropout was employed to all representation layers before classification: POS tag, dependency arc and label with the recommended rate of 0.33; we used a fixed dimension of 100 for dependency arc and label embeddings [24].

Hyperparameters	Range	Best
Batch size	[10, 32]	20
Dropout input	[0.1, 0.5]	0.34
Dropout GCN	[0.1, 0.5]	0.19
Dropout classification	0.33	0.33
Learning rate	Default	0.001
Gradient clipping	[5, 30]	10
Weight decay (L2)	[1e-2, 1e-5]	1e-4
Dim. of POS	[16, 50]	16
Dim. of dependency	100	100

Table 3 presents word representations used in our experiments as well as training data and syntactic parsers for our SIWR model. In our experiments, these settings are chosen depending on the domain. The static embeddings used in our experiments including **W2V** (200d) was trained with word2vec [34] from Wikipedia 2015 [11]^5^; **GloVe** (100d) was trained on Wikipedia 2014 and Gigaword 5 [18]^6^ and **PubMed** (200d) was trained on MEDLINE/PubMed 2018 using word2vec [34].^7^ Regarding contextualised representations, we used the small version of ELMo (256d) released by Peters et al. [5].^8^

**Table 3:** Word representations, training data and dependency parsers that are used in our experiments.

Task		Nested NER and RE	*N*-ary RE
**Static Embeddings**		W2V	GloVe
**SIWRs**	Base	ELMo	PubMed
	Data	1B Word	PubMed
	Parser	Stanford CoreNLP	ScispaCy

As shown in Table 3, we used **SIWRs**_**ELMo**_ (the enriched ELMo) on general data for the ACE2005 dataset, resulting in two tasks: nested NER and binary RE. To obtain SIWRs_ELMo_, around 0.2% of the one billion word benchmark [35], which was also used by ELMo, was randomly selected and automatically parsed using Stanford CoreNLP [16].^10^ The processed data was divided into two parts for training (30 k) and development (30 k). We then used the training set to train our SIWRs_ELMo_. As ELMo was trained on the general domain, the contexts that it captures are likely to be biased towards general language. To address this bias, we used PubMed as our base representations for the *n*-ary RE task (**SIWRs**_**PubMed**_). We enrich PubMed using our model by pre-training on a PubMed subset containing the same number of sentences while enriching ELMo. We also used ScispaCy which is a syntactic parser trained on biomedical data [17] to obtain dependencies.^11^

For each type of base representations, once our model was trained, we extracted SIWRs on automatically parsed evaluation data without any further fine-tuning efforts. To ensure a controlled comparison, we fixed the neural architecture for each downstream task. Since nested NER and binary RE are based on general domain, our SIWR model was trained once and used in both evaluations to extract SIWRs. We kept all hyperparameters to be the same in the reported papers, except for the *n*-ary model, for which we found that due to the masked entity word form, it is more difficult to capture the context with many masked tokens. Hence, we increased the learning rate to speed up the learning process. The large learning rate may cause the model more easily over-fitting to the training data. We prevented this by adding a small value of weight decay along with a word dropout rate. Other hyperparameters remained the same as reported in Peng et al. [29].

The current trend of using large pre-trained models e.g., BERT [7], might question the need for explicit syntactic information. BERT demonstrated that fine-tuning the entire model can perform better than using pre-trained word representations. To compare with such fine-tuning methods and to show the role of explicitly including syntactic structures, we performed experiments using BERT as base representation and fine-tuned it for nested NER and binary RE tasks.^9^ We employed the pre-trained BERT base model (768d) in our experiments. To use BERT as base representation, we extracted the intermediate embeddings from the last four hidden layers of BERT as these layers were reported to be the best performing representations [7]. Due to memory limit, we further added a feed-forward hidden layer with 256 dimensions on top of BERT contextual representations to reduce the representation size when applying them in the downstream linguistic tasks. While all the non-BERT training and experiments were done on a Tesla K20 (5 GB GPU memory and CUDA compute capability 3.5), the experiments with BERT were conducted using a GTX 1080 Ti (11 GB memory) due to its computational requirements.

### 4.2 Baseline settings

Table 4 presents the hyperparameters for the baseline models used in our experiments on NER, binary and *n*-ary RE, respectively. For nested NER and binary RE, we employed the baseline models using Chainer library [36]. Meanwhile, the *n*-ary model was implemented with Theano framework [32]. We do not change hyperparameters in nested NER and binary RE, which allow us to do a controlled comparison with base representations. *N*-ary RE data, however, was difficult to train on and dependent on precise hyperparameter tuning since it was automatically constructed using a distant supervision approach. We then fine-tuned the hyperparameters of the *n*-ary baseline. Given an identical base architecture across representations for each task, we can then attribute any difference in performance to the proposed SIWRs over base representations.

**Table 4:** Hyperparameters for the nested named recognition [25] (Table 4a), binary relation extraction model with up to 4 length walks [27] (Table 4c) and and *N*-ary relation extraction model [29] with PubMed as the base representations for SIWRs (Table 4b).

(a) Nested NER		
**Hyperparameters**	**Original/ ELMo** & **SIWRs**	
Batch size	91	
No. of hidden units	200/ 256	
Dim. of char. emb.	28	
Dropout rate	0.1708	
Learning rate	0.00426	
Gradient clipping	11	
Weight decay (L2)	9.43e-5	

**(b)***N***-ary RE**		

**Hyperparameters**	**PubMed**	**SIWRs**
Batch size	8	15
LSTM dimension	150	150
Learning rate	0.02	0.3735
Weight decay (L2)	0.0	0.0021
Word dropout rate	0.0	0.0019

**(c) Binary RE**		

**Hyperparameters**	**Original/ ELMo** & **SIWRs**	
Unk. word prob	0.01/ 0.0	
Batch size	10	
Word dimension	200/ 256	
Position dimension	25	
Type dimension	20	
LSTM dimension	100	
Pair dimension	100	
β	0.77	
Input dropout rate	0.11	
Output dropout rate	0.32	
Learning rate	0.002	
Weight decay (L2)	0.000057	
Gradient clipping	24.4	

## 5 Benchmark results

We first summarise the main results compared to base representations, then compare our SIWRs with fine-tuning the large pre-trained model BERT. We also discuss the performance of our method on each evaluation task.

Table 5 depicts the performance of SIWRs on the three information extraction tasks in comparison with the baseline models using static embeddings (Static) and ELMo. For *n*-ary RE, unlike other tasks, we employed the first layer of ELMo as the input representations. The performance of the same setting with other tasks is lower than the first layer, 70.6 and 75.8% accuracy, respectively. Adding SIWRs to the baselines improves the performance from 3% to 9% relative points compared to the base representations, i.e., SIWRs_ELMo_ versus ELMo for nested NER and binary RE, and SIWRs_PubMed_ versus Static/PubMed for *n*-ary RE.

**Table 5:** Test set results with different embeddings over three NLP tasks. We use task-related evaluation metrics: F1-score (%) for nested NER, and RE and accuracy (%) for *n*-ary RE. The “Improvement” column lists the absolute and relative improvements over the base representations of SIWRs, i.e., SIWRs_ELMo_ for nested NER and RE and SIWRs_PubMed/Static_ for *n*-ary RE.

Method		NER				Binary RE				*N*-ary RE
		P	R	F1		P	R	F1		Acc.
Static		79.54	64.55	71.26		68.77	60.64	64.45		77.1
ELMo		**80.17**	72.12	75.93		**70.48**	61.60	65.74		75.8
SIWRs_base_		79.57	**75.61**	**77.54**		69.74	**64.47**	**67.00**		**79.3**
Improvement (Absolute/ Relative)		–	–	1.6/ 6.64%		-	-	1.3/ 3.79%		2.2/ 9.6%

Furthermore, we compare the performance of using contextual embeddings as well as fine-tuning BERT in Table 6. With contextual embeddings, we add only *k* scalar weights rather than fine-tuning large pre-trained LM ($\theta_{\mathrm{LM}}$) in downstream tasks ($k\ll \theta_{\mathrm{LM}}$). Although fine-tuning BERT for nested NER yields slightly better performance than our SIWRs_BERT_, our method employs computational benefits, in that the computationally expensive representations of the training data are pre-computed only once. After getting the representations, many experiments using less computationally intensive model architectures can be run on top of these representations. Moreover, our results indicate that pre-trained embeddings can perform better than fine-tuning in terms of binary RE. This may be due to the complexity of BERT compared to the small data size of the task, and the prerequisite syntactic information embedded in SIWRs. In other words, fine-tuning may require a task-specific model design to be added in order to fit downstream linguistic tasks.

**Table 6:** Comparison of contextual representations and fine-tuning large-scale language model. Text subscription indicates the base representations used in SIWRs.

Method	Nested NER								RE						
	DEV				TEST				DEV				TEST		
	P	R	F1		P	R	F1		P	R	F1		P	R	F1
	**Static Embeddings**														
Baseline	78.10	65.14	71.03		79.54	64.55	71.26		64.51	64.28	64.39		68.77	60.64	64.45
	**Contextual Embeddings**														
ELMo	80.97	72.88	76.71		80.17	72.12	75.93		66.19	61.80	63.92		70.48	61.60	65.74
SIWRs_ELMo_	77.37	72.95	75.10		79.57	75.61	77.54		65.96	65.61	65.78		69.74	64.47	67.00
BERT–feature	80.40	79.78	80.09		81.49	80.02	80.75		68.10	70.03	69.05		71.59	69.85	70.71
SIWRs_BERT_	81.12	79.51	80.31		83.32	80.84	82.06		**68.27**	**71.35**	**69.78**		**73.78**	**71.16**	**72.45**
	**Fine-tuning LM**														
BERT–fine-tuning	**82.96**	**82.71**	**82.83**		**84.24**	**81.49**	**82.84**		63.37	65.78	64.56		66.02	67.68	66.84

In particular, we compared the performance of our nested NER baseline with different base representations and the current state-of-the-art models in Table 7. While stacked BiLSTM-CRF [25] is the baseline that we employed, the SOTA (Merge and Label) performance with different embeddings were reported in Fisher and Vlachos [26]. As shown in Table 7, our SIWRs consistently improved the performance over base representations. Although our baseline is around 2% points lower than the SOTA in terms of F1-score using either static or contextual embeddings, i.e, ELMo and contextual BERT (BERT-feature), we improved the performance of the baseline by adding syntactic information and the results were then comparable to the SOTA. We analyse the performance gain in §6.4.

**Table 7:** Performance comparison on Nested NER – ACE 2005 test set. Text subscription indicates the base representations used in SIWRs. +E denotes ELMo is used as base representations, +B-f is BERT-feature, +B-ft is BERT-fine-tune, +S_E_ and +S_B_ are SIWRs with ELMo or BERT as base representations, respectively.

	Previous work					Ours					
	[25] Stacked BiLSTM-CRF	[26] Merge & Label				[25] Baseline–Stacked BiLSTM-CRF					
		GloVe	+E	+B-f		W2V	+E	+S_E_	+B-ft	+B-f	+S_B_
**P**	74.2	75.1	79.7	82.7		79.54	80.17	79.57	84.24	81.49	83.32
**R**	70.3	74.1	78.0	82.1		64.55	72.12	75.61	81.49	80.02	80.84
**F1**	72.2	74.6	78.9	82.4		71.26	75.93	77.54	82.84	80.75	82.06

We also report the performance of SIWRs compared with other representations used in binary relation extraction task (Table 8). SIWRs improved the performance of the baseline model using ELMo to 67% F1-score (1.3% points of overall performance). Compared to that, fine-tuning BERT, in this case, is less effective than our method, with comparable performance when we enriched ELMo but a much higher computational cost (see §6.5 for analysis). Furthermore, we show the new reported SOTA performance by using BERT as our base representations (SIWRs_BERT_) with 72.45% in F1-score. This indicates that syntactic information is helpful even when using large-scale pre-trained models. We also show the performance of a recent model – CNN + RL proposed by Ye et al. [28] that uses a ranking loss for relation classification. Unlike our experimental setting, they did not consider directionality when generating relation candidates (i.e., relation from the first to the second argument or vice versa) in the experiments on the English part of ACE 2005. Not considering relation direction makes the task easier, hence they got higher performance than our baseline. This performance gap can be resolved by our SIWRs which provide syntactic dependencies to infer relation direction.

**Table 8:** Binary relation extraction performance comparison on ACE2005 test set. Text subscription indicates the base representations used in SIWRs. +E denotes ELMo is used as word representations, +B-f is BERT-feature, +B-ft is BERT-fine-tune, +S_E_ and +S_B_ are SIWRs with ELMo or BERT as base representations respectively.

Model	Previous work					Ours					
	[11] SPTree	[27] Walk-based	[28] CNN + RL			[27] Baseline – Walk-based					
			Base	+BIO Tag		W2V	+E	+S_E_	+B-ft	+B-f	+S_B_
**P**	70.1	69.7	58.8	61.3		68.77	70.48	69.74	66.02	71.59	**73.78**
**R**	61.2	59.5	57.3	**76.7**		60.64	61.60	64.47	67.68	69.85	71.16
**F1**	65.3	64.2	57.2	67.4		64.45	65.74	67.00	66.84	70.71	**72.45**

Last, the results on *n*-ary relation extraction task are shown in Table 9, where the effectiveness of our model in capturing syntactic information is demonstrated. We observe that ELMo did not perform well in this domain-specific task compared to static word representations. Hence, we included an experiment using the first layer representations from ELMo which are the combination of character and static word representations. Surprisingly, the static representations from ELMo performed comparably to the baseline model using GloVe. Meanwhile, using domain-specific word representations (PubMed) produced better results than general domain word representations. The baseline performance of using PubMed is comparable to the GraphLSTM model which included syntactic information. We followed previous work and used the general domain parser – Stanford CoreNLP. Our SIWRs outperformed the GraphLSTM by 0.9% and the base representations setting by 1.54% in terms of accuracy. We additionally applied the in-domain parser – ScispaCy to the dataset. As expected, our SIWRs further improved the performance to 79.3% accuracy. This indicates that our method can enrich word representations with both in- and out-of-domain parsers.

**Table 9:** *N*-ary relation extraction accuracy on the drug-gene-mutation data [29]. LSTM and GraphLSTM were reported results from [29].

Model	Peng et al. (2017) [29]			Ours – LSTM					
	LSTM	GraphLSTM		PubMed	ELMo			SIWRs_PubMed_	
					all	1st		CoreNLP	ScispaCy
**Accuracy**	75.3	77.9		77.2	70.6	75.8		78.7	**79.3**

## 6 Analysis

We analysed our model including the different base representations, the amount of pre-training data, the breakdown contributions of each component, the need for syntax in the advent of large pre-trained models, the number of model parameters and the computational environment.

### 6.1 Comparison between different base representations

Our model is not restricted to ELMo; Table 10 shows the flexibility of our model with several word representations including static and contextualised ones: W2V, GloVe, and ELMo. W2V and GloVe are static embeddings with the dimensionality of 200, while ELMo is contextualised embeddings with the dimensionality of 256 and 3 layers. As shown in the table, we obtained 1–4% absolute improvements in F1-score compared to the original representations. Although ELMo already included contextual and statistically inferred syntactic features [5], our SIWR model provides explicit syntactic information to the embeddings. GloVe obtained the most significant improvement of 4.35% absolute F1-score and a relative error reduction of 13.84%. The distinct improvement over the static embeddings, i.e., GloVe, may be partly due to the embedded context. Our results show that incorporating syntactic information is useful in deep neural models.

**Table 10:** Nested named entity recognition results on ACE2005 development set with different base representations and their enriched alternatives.

Partition	Embedding	W2V	GloVe	ELMo
Dev	Original	71.03	66.31	76.71
	SIWRs	71.00	71.86	75.09
Test	Original	71.26	68.58	75.93
	SIWRs	72.67	72.93	77.54
Improvements (Absolute/ Relative)		1.41/ 4.91%	4.35/ 13.84%	1.61/ 6.68%

### 6.2 Effects of the number of pre-training samples

**Fig. 3:**
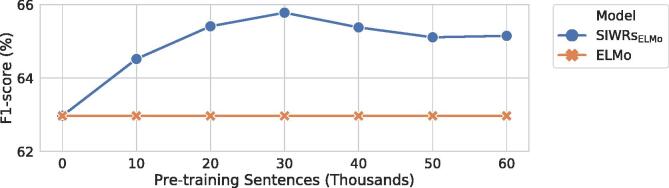
Binary relation extraction performance of SIWRs_ELMo_ with different numbers of pre-training sentences on the ACE2005 development set. 60 k corresponds to about 0.2% of the 1B Word dataset in §4. The exact numbers of instances used in Fig. 3 are: 10,253 (10 k); 20,506 (20 k); 30,759 (30 k); 41,012 (40 k); 51,265 (50 k); 61,326 (60 k).

We show the performance of SIWRs_ELMo_ with different numbers of pre-training sentences on the binary RE development set in Fig. 3. The learning curve reveals that only a relatively modest amount of pre-training sentences, i.e., 10 k, is enough to improve the performance of downstream tasks and that this performance is almost stable after the 10 k sentences. This figure shows that it is reasonable that we chose 30 k sentences for pre-training.

### 6.3 Ablation studies

We investigate the contributions and effects of the various components in our proposed model by evaluating the binary RE performance using SIWRs_ELMo_ on the ACE2005 development set (Table 11).

• **–POS** (POS tagging): We re-trained our model without POS tagging by considering only dependency parsing when computing loss.
• **–DP** (Dependency Parsing): Contrary to –POS, we retrained our model without dependency parsing loss and retained POS tagging.
• **–SO** (Sequential Order): We removed sequential order connection in GCN to measure its importance to the learning process.
• **–SI** (Self-Information): We removed self-information connection in GCN to see if a word can obtain its information only from the dependency context.

**Table 11:** Binary relation extraction performance of ablated SIWRs variants on ACE2005 development set, i.e., without POS tagging, dependency parsing, sequential information and self-information in GCN encoder. † denotes significance at p<0.05 compared to ELMo.

Model	P	R	F1
SIWRs_ELMo_	65.96	65.61	65.78^†^
–POS	66.36	64.01	65.17
–DP	67.30	59.86	63.36
–SO	66.48	61.72	64.01
–SI	66.86	62.25	64.47
ELMo	66.19	61.80	63.92

As we observe in Table 11, each removal of different components of our model substantially reduced the performance. We performed the Approximate Randomisation test [37] on the results to measure the difference among different ablations. The full setting of SIWRs_ELMo_ (POS, DP, SO and SI) is significantly different from ELMo with p<0.05. This observation indicates the importance of selected information added in our model along with our learning objectives in order to incorporate syntactic information into word representations. We also showed that merely adding randomly-initialised parameters is not enough to get better performance, since when removing each component, the significance test shows no significant difference compared to ELMo.

**Fig. 4:**
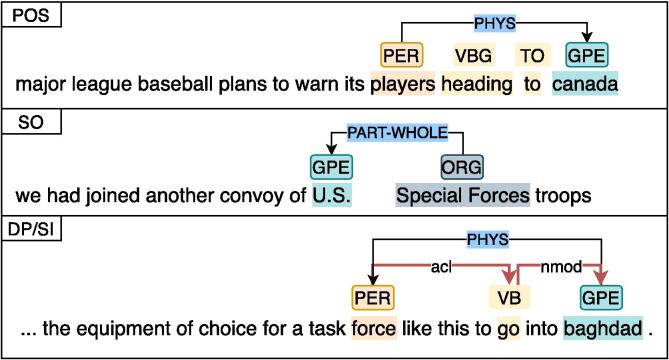
Missing predictions when removing a component, which is named in white box on the top left corner, compared to SIWRs_ELMo_. Other POS tags, dependency connections and relations are obmitted for simplicity.

However, we observe that the precision is better when removing a component at a time in contrast to the decrease in recall. To explain this phenomenon, we randomly check some of the different false negative errors between the ablations and SIWRs_ELMo_. We identify the most frequent error for each ablation as shown in Fig. 4. In the first case, the POS sequence between two entities can also signal relationships, e.g., *PER* VB [IN—TO] (DT) *GPE*. Meanwhile, sequence order (SO) along with POS reveals relationships without a specific textual trigger, e.g., *U.S.* and *Special Forces* in the second case. In the last case of Fig. 4, syntactic dependency structure of a sentence provides helpful clues for RE [11], self-information (SI) reveals a helpful multi-hop dependency path between entities by capturing direct dependent words in the first GCN layer and the multi-hop in the second GCN layer. In general, the patterns from the components generally signal more relationships between entities but also include more noises. These noises thus lead to the drop in precision while increasing the recall.

### 6.4 Impact of syntactic information

Table 12 presents the nested NER performance of using ELMo versus SIWRs_ELMo_ on the ACE 2005 development set over different named entity categories. The categories are already sorted in descending order according to the numbers of gold annotations (the same trend is observed in the training data). In general, SIWRs_ELMo_ significantly outperformed ELMo in five out of the seven categories and particularly in the rare ones. Although the overall micro F1-score of using ELMo is higher than using SIWRs_ELMo_ on the development set, when applying on the test set, SIWRs_ELMo_ got higher performance than ELMo with 67.00% and 65.74%, respectively). This behaviour may be attributed to the POS tag information embedded in SIWRs_ELMo_ which provides additional clues for extracting named entity in cases of lack of training instances or on unseen data.

**Table 12:** Nested named entity recognition performance of using ELMo and SIWRs_ELMo_ on ACE2005 development set.

		ELMo				SIWRs_ELMo_		
		P	R	F1		P	R	F1
PER		**84.89**	**82.85**	**83.86**		82.99	81.89	82.44
GPE		**80.31**	**73.87**	**76.96**		79.37	72.84	75.97
ORG		**75.58**	54.28	63.18		67.59	**60.96**	**64.11**
WEA		61.36	57.45	59.34		**73.33**	**58.51**	**65.09**
FAC		**63.24**	51.81	56.95		56.70	**66.27**	**61.11**
LOC		62.75	39.51	48.49		**63.16**	**44.44**	**52.17**
VEH		**73.47**	44.44	55.39		63.49	**49.38**	**55.56**
Micro		**80.97**	72.88	**76.71**		78.05	**73.96**	75.95
Macro		**71.66**	57.74	63.45		69.52	**62.04**	**65.21**

**Fig. 5:**
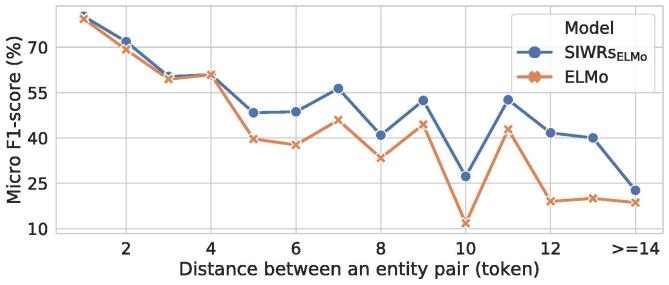
Comparison of the binary relation extraction performance on entity pairs with different distances on ACE2005 development set using SIWRs_ELMo_ and ELMo.

**Fig. 6:**
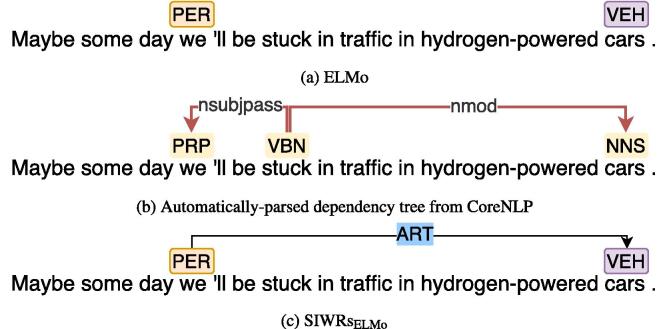
Relation prediction examples from ACE2005 dataset and the automatically parsed tree. Orange and purple rectangles denote two different entity types (*PER* for person and *VEH* for vehicle respectively), while yellow rectangles denote part-of-speech tags. Red arrow connections denote dependency relations between words, while the relation between entities is illustrated by a black arrow with its type in blue background. *ART* stands for artifact. Other dependency connections and relations are obmitted for simplicity.

To evaluate the benefits of dependency information, we further analyse the binary RE performance of SIWRs_ELMo_ and ELMo in capturing distantly-related entity pairs on the ACE2005 development set. Fig. 5 shows that our SIWRs_ELMo_ performed better than ELMo in capturing long-distance context. As it can be observed, for entity pairs with a distance shorter than 5, the performance of using ELMo and SIWRs_ELMo_ are nearly the same. The performance gap then gets wider when the distance between entities is longer. The performance gain using SIWRs_ELMo_ can be attributed to dependency-based context information, which is useful for detecting relations between distant entities. We present an example of such case in Fig. 6. The figure illustrates that using only ELMo could not capture the relation between *PER* (“*we*”) and *VEH* (“*cars*”). Using SIWRs_ELMo_, the downstream model was then able to detect the relation by leveraging dependency connections. However, when the distance is longer than 14, the performance of using SIWRs_ELMo_ drops, close to using ELMo (Fig. 5). This may be partly because the distance longer than 14 is beyond the dependency length. In future work, we will include coreference, as well as discourse connections to address long-distance dependencies.

### 6.5 Computaional cost

Table 13 compares the numbers of parameters used for training different representations and applying them on downstream NLP tasks. The table also shows the parameters used for training SIWRs with different base representations as well as applications to downstream tasks. In general, with contextual representations, we require fewer parameters for training in downstream models even compared to static word representations. More precisely, our SIWRs requires L+2 scalar numbers to be trained when applied in NLP models compared to the whole model parameters in fine-tuning methods, i.e., $\theta_{\mathrm{BERT}}$ where $L+2\ll \theta_{\mathrm{BERT}}$. Most of the current work on adding a syntactic inductive bias into word representations or large pre-trained models requires re-training the language models [38], [15], [14]. By contrast, we need to train the SIWR model, but we keep the computational costs down because we do not need to adapt the available well-pre-trained models. These training-from-scratch methods are costly when computational resources are limited.

**Table 13:** Pre-trained model parameters and downstream trainable parameters.

		Original	Downstream	SIWRs	Downstream SIWRs
word2vec		$|V|\times d_{w}$	$+(|V|+1)\times d_{w}$	$\theta_{\mathrm{SIWR}}$	+(1+2)
GloVe					
PubMed					
ELMo		$\theta_{\mathrm{ELMo}}$	+L		+(L+2)
BERT		$\theta_{\mathrm{BERT}}$	$\theta_{\mathrm{BERT}}-\theta_{\mathrm{MaskedLM}}-\theta_{\mathrm{NSP}}$		

In particular, since the number of parameters for training the SIWR model is relatively small compared to BERT, we trained our model on a Tesla K20 (5 GB GPU memory and CUDA compute capability 3.5). Other downstream models using static, contextual and SIWRs are also trained using a Tesla K20. In contrast, as BERT required large memory for fine-tuning, we ran its corresponding models on a GTX 1080 Ti (11 GB memory) for every downstream task. In this case, contextual representations and our SIWRs require less computational resources than the fine-tuning method (BERT). Although our model is not computationally expensive, our SIWRs can boost the performance of downstream linguistic tasks to be competitive with the fine-tuning model. This finding opens a potential direction to investigate the role of syntactic information and other rich linguistic information in general.

## 7 Related work

### 7.1 Word representations and pre-trained models

Standard word representation approaches compress all contexts corresponding to a single word into a continuous vector. Word representations exhibit bias in written language, i.e., a word representation often biases towards its frequent context. To address this bias, several methods have been proposed, such as sense-specific embeddings that treated each sense as a new word [39], [40], [41]. However, sense-specific embeddings require prior knowledge of word sense in a given context.

Contextualised word representation models have been proposed to represent a word within a context [4], [5]. These models have achieved impressive results when applied to several natural language understanding problems. The models were pre-trained on large-scale data available tasks, such as language modelling and machine translation. They derived internal representations of these models to form contextual representations.

Alternatively, recent large-scale pre-trained LMs such as Open GPT [6], BERT [7] and GPT2 [8] have been used in downstream NLP applications with fine-tuning methods. BERT [7], as the current state-of-the-art fine-tuning model, is trained with language modelling objectives (masked language modelling and next sentence prediction) that explore statistical knowledge in a large amount of data. To apply it in a downstream task, parameters of the whole pre-trained model instead of the last layer are typically learned and adapted to a target task. Therefore, this leads to an incremental requirement regarding computation resources, i.e., GPUs with high memory. Our method, however, adopted the contextual word representation scheme for downstream applications. The number of additional hyperparameters that need to be trained in downstream models is only a few scalar numbers, i.e., $L+2\ll \theta_{\mathrm{LM}}$.

### 7.2 Syntactically related work

Syntactic information have been exploited in the age of deep neural models [42], [43], [44], [45], [46]. However, the amount of work using syntactic information is relatively few compared to the ones without syntactic information. Recent work by Strubell et al. [47] attempted to incorporate syntax by adding a restricted attention layer that attends to the syntactic parent of a word into their model. Our method, instead, introduces syntactic information into NLP models simply by replacing the word representations without explicitly changing their architecture. Moreover, there is not a standard framework to incorporate syntactic information. Similar to word embeddings, our method provides a general common input to transfer in several tasks without changing model architecture for every single task. Pre-incorporating syntactic information also means much fewer parameters needed to be optimised on downstream tasks.

Many methods have been proposed for incorporating linguistic constraints, i.e., syntactic and lexical knowledge, to static embeddings [48], [49], [50], [51]. With the advent of pre-trained LMs, the NLP community is also revisiting the role of linguistic knowledge in such models [38], [14], [15]. However, these methods require training from scratch in a multitask learning setting, which is computationally expensive and time-consuming. On the contrary, our method needs less computational resources by pre-computing word representations from existing pre-trained models and enriching them.

### 7.3 Graph neural networks

Syntactic dependencies are essentially a directed graph where words constitute the nodes and edges correspond to the dependencies between words. GCNN was firstly proposed by Kipf and Welling [52] and applied on citation networks and knowledge graph datasets. It was later used for semantic role labelling [22], multi-document summarization [53] and relation extraction [54]. Similar to previous work, our GCN was trained over a dependency tree along with self-information connections. We extend GCN with the sequential order of text as word order is certainly important in English. Our GCN is used as an encoder, and we decode the dependencies while simultaneously predicting POS tag. The closest work to ours is by Vashishth et al. [38] who learned word embeddings using GCN as the core of a LM. They trained a neural LM from plain text with dependencies and lexical structures that compress all meanings of a word into a single vector, including polysemy. By contrast, our work is based on existing pre-trained LMs which uses much fewer data compared to training from scratch. The resulting SIWRs can improve downstream applications simply by replacing the word representations in these models.

Self-attention has gained special attention from the NLP community. Recent related work [19], [55] attempted to map self-attention heads to syntactic related information, they showed that these heads are able to capture the structural properties of English. However, Strubell et al. [13] showed that self-attention needs explicit syntactic information. They improved the performance over vanilla self-attention by forcing the model to attend on the dependency head and predict POS tag in a multitask learning setting. Compatible with their findings, our experiments showed that explicitly integrating syntactic information is empirically beneficial for BERT [7], i.e., a self-attention based LM, in terms of binary relation extraction in our experiments (Table 6).

## 8 Conclusion

We proposed a novel way to include syntactic information to word representations. The enhanced representations are called syntactically-informed word representations (SIWRs). SIWRs was obtained by training a graph-based model to capture two types of syntactic information on the data, POS, and dependencies. Our method is the first attempt to include such syntactic information without retraining language models by leveraging existing well-pre-trained models. The computational resource and cost-effectiveness of our syntactically informative method are that fewer computational resources and less time are needed to train our model compared to previous methods. We empirically demonstrated the contributions of including syntactic information in our experiments. In particular, SIWRs achieved gains over the base representations, i.e., ELMo and PubMed, on three NLP tasks: nested NER, binary RE and *n*-ary RE, and improved performance on these tasks baselines. We also demonstrated the flexibility and effectiveness of SIWRs by showing improvements over different pre-trained representations. Meanwhile, we found that our SIWRs performed better than BERT in language understanding tasks that required inference capability, i.e., binary RE.

Although we only evaluated our SIWR model on information extraction tasks, our method can be applied to any other downstream tasks. For future research, we will investigate other linguistic structures such as semantic roles. An information mask similar to masked LM in Devlin et al. [7] will also be helpful to embed input information. We hope that this study will attract more investigation in the NLP community to revisit the incorporation of rich linguistic information in NLP tasks.

## Declaration of Competing Interest

The authors declare that they have no known competing financial interests or personal relationships that could have appeared to influence the work reported in this paper.

## CRediT authorship contribution statement

**Thy Thy Tran:** Conceptualization, Methodology, Writing - original draft, Writing - review & editing, Visualization. **Makoto Miwa:** Writing - review & editing, Supervision. **Sophia Ananiadou:** Writing - review & editing, Supervision.

## Footnotes

1 https://github.com/meizhiju/layered-bilstm-crf.

2 This is the latest work on sentential binary relation extraction at the time we conducted this experiment.

3 http://hanover.azurewebsites.net.

4 https://github.com/hyperopt/hyperopt.

5 http://tti-coin.jp/data/wikipedia200.bin.

6 http://nlp.stanford.edu/projects/glove/.

7 http://nlp.cs.aueb.gr/software.html.

8 https://allennlp.org/elmo.

10 We use universal dependencies from Stanford CoreNLP version 3.9.1.

11 We use universal dependencies from ScispaCy version 0.2.0.

9 We did not conduct experiments on *n*-ary RE as there is no available BERT implementation in Theano framework, in which the *n*-ary RE model was developed.

## References

[1] Arora S., Li Y., Liang Y., Ma T., Risteski A. (2016). A latent variable model approach to pmi-based word embeddings. Trans. Associat. Comput. Linguist..

[2] J. Tissier, C. Gravier, A. Habrard, Dict2vec: Learning word embeddings using lexical dictionaries, in: Conference on Empirical Methods in Natural Language Processing (EMNLP 2017), 2017, pp. 254–263.

[3] X. Xin, F. Yuan, X. He, J.M. Jose, Batch is not heavy: Learning word representations from all samples, in: Proceedings of the 56th Annual Meeting of the Association for Computational Linguistics (Volume 1: Long Papers), Association for Computational Linguistics, 2018, pp. 1853–1862.

[4] B. McCann, J. Bradbury, C. Xiong, R. Socher, Learned in translation: Contextualized word vectors, in: Advances in Neural Information Processing Systems, 2017, pp. 6294–6305.

[5] M. Peters, M. Neumann, M. Iyyer, M. Gardner, C. Clark, K. Lee, L. Zettlemoyer, Deep contextualized word representations, in: Proceedings of the 2018 Conference of the North American Chapter of the Association for Computational Linguistics: Human Language Technologies, Volume 1 (Long Papers), Association for Computational Linguistics, New Orleans, Louisiana, 2018, pp. 2227–2237.

[6] A. Radford, K. Narasimhan, T. Salimans, I. Sutskever, Improving language understanding with unsupervised learning, Technical Report, Technical report, OpenAI, 2018.

[7] J. Devlin, M.-W. Chang, K. Lee, K. Toutanova, BERT: Pre-training of deep bidirectional transformers for language understanding, in: Proceedings of the 2019 Conference of the North American Chapter of the Association for Computational Linguistics: Human Language Technologies, Volume 1 (Long and Short Papers), Association for Computational Linguistics, Minneapolis, Minnesota, 2019, pp. 4171–4186. URL https://www.aclweb.org/anthology/N19-1423. doi:10.18653/v1/N19-1423.

[8] Radford A., Wu J., Child R., Luan D., Amodei D., Sutskever I. (2019). Language models are unsupervised multitask learners. OpenAI Blog.

[9] C. Alt, M. Hübner, L. Hennig, Fine-tuning pre-trained transformer language models to distantly supervised relation extraction, in: Proceedings of the 57th Annual Meeting of the Association for Computational Linguistics, Association for Computational Linguistics, Florence, Italy, 2019, pp. 1388–1398. URL https://www.aclweb.org/anthology/P19-1134. doi:10.18653/v1/P19-1134.

[10] H. Wang, M. Tan, M. Yu, S. Chang, D. Wang, K. Xu, X. Guo, S. Potdar, Extracting multiple-relations in one-pass with pre-trained transformers, in: Proceedings of the 57th Annual Meeting of the Association for Computational Linguistics, Association for Computational Linguistics, Florence, Italy, 2019, pp. 1371–1377. URL https://www.aclweb.org/anthology/P19-1132. doi:10.18653/v1/P19-1132.

[11] M. Miwa, M. Bansal, End-to-end relation extraction using lstms on sequences and tree structures, in: Proceedings of the 54th Annual Meeting of the Association for Computational Linguistics (Volume 1: Long Papers), Association for Computational Linguistics, 2016, pp. 1105–1116. doi:10.18653/v1/P16-1105.

[12] Kuncoro A., Dyer C., Hale J., Yogatama D., Clark S., Blunsom P. (2018). Lstms can learn syntax-sensitive dependencies well, but modeling structure makes them better. Proceedings of the 56th Annual Meeting of the Association for Computational Linguistics (Volume 1: Long Papers).

[13] E. Strubell, P. Verga, D. Andor, D. Weiss, A. McCallum, Linguistically-informed self-attention for semantic role labeling, in: Proceedings of the 2018 Conference on Empirical Methods in Natural Language Processing, Association for Computational Linguistics, 2018, pp. 5027–5038.

[14] A. Lauscher, I. Vulić, E.M. Ponti, A. Korhonen, G. Glavaš, Informing unsupervised pretraining with external linguistic knowledge, arXiv preprint arXiv:1909.02339 (2019).

[15] H. Peng, R. Schwartz, N.A. Smith, Palm: A hybrid parser and language model, arXiv preprint arXiv:1909.02134 (2019).

[16] C.D. Manning, M. Surdeanu, J. Bauer, J. Finkel, S.J. Bethard, D. McClosky, The Stanford CoreNLP natural language processing toolkit, in: Association for Computational Linguistics (ACL) System Demonstrations, 2014, pp. 55–60.

[17] M. Neumann, D. King, I. Beltagy, W. Ammar, Scispacy: Fast and robust models for biomedical natural language processing, CoRR abs/1902.07669 (2019). arXiv:1902.07669

[18] Pennington J., Socher R., Manning C. (2014). Glove: global vectors for word representation. Proceedings of the 2014 Conference on Empirical Methods in Natural Language Processing (EMNLP).

[19] K. Clark, U. Khandelwal, O. Levy, C.D. Manning, What does BERT look at? an analysis of BERT’s attention, in: Proceedings of the 2019 ACL Workshop BlackboxNLP: Analyzing and Interpreting Neural Networks for NLP, Association for Computational Linguistics, Florence, Italy, 2019, pp. 276–286. URL https://www.aclweb.org/anthology/W19-4828. doi:10.18653/v1/W19-4828.

[20] S. Swayamdipta, M. Peters, B. Roof, C. Dyer, N.A. Smith, Shallow syntax in deep water, arXiv preprint arXiv:1906.04341 (2019).

[21] R. McDonald, G. Brokos, I. Androutsopoulos, Deep relevance ranking using enhanced document-query interactions, in: Proceedings of the 2018 Conference on Empirical Methods in Natural Language Processing, Association for Computational Linguistics, 2018, pp. 1849–1860.

[22] D. Marcheggiani, I. Titov, Encoding sentences with graph convolutional networks for semantic role labeling, in: Proceedings of the 2017 Conference on Empirical Methods in Natural Language Processing, Association for Computational Linguistics, 2017, pp. 1506–1515.

[23] D. Marcheggiani, J. Bastings, I. Titov, Exploiting semantics in neural machine translation with graph convolutional networks, in: Proceedings of the 2018 Conference of the North American Chapter of the Association for Computational Linguistics: Human Language Technologies, Volume 2 (Short Papers), Association for Computational Linguistics, 2018, pp. 486–492.

[24] Dozat T., Manning C.D. (2016). Deep biaffine attention for neural dependency parsing. ICLR.

[25] M. Ju, M. Miwa, S. Ananiadou, A neural layered model for nested named entity recognition, in: Proceedings of the 2018 Conference of the North American Chapter of the Association for Computational Linguistics: Human Language Technologies, Volume 1 (Long Papers), volume 1, 2018, pp. 1446–1459.

[26] J. Fisher, A. Vlachos, Merge and label: A novel neural network architecture for nested NER, in: Proceedings of the 57th Annual Meeting of the Association for Computational Linguistics, Association for Computational Linguistics, Florence, Italy, 2019, pp. 5840–5850. URL https://www.aclweb.org/anthology/P19-1585. doi:10.18653/v1/P19-1585.

[27] F. Christopoulou, M. Miwa, S. Ananiadou, A walk-based model on entity graphs for relation extraction, in: Proceedings of the 56th Annual Meeting of the Association for Computational Linguistics (Volume 2: Short Papers), vol. 2, 2018, pp. 81–88.

[28] W. Ye, B. Li, R. Xie, Z. Sheng, L. Chen, S. Zhang, Exploiting entity BIO tag embeddings and multi-task learning for relation extraction with imbalanced data, in: Proceedings of the 57th Annual Meeting of the Association for Computational Linguistics, Association for Computational Linguistics, Florence, Italy, 2019, pp. 1351–1360. URL https://www.aclweb.org/anthology/P19-1130. doi:10.18653/v1/P19-1130.

[29] Peng N., Poon H., Quirk C., Toutanova K., tau Yih W. (2017). Cross-sentence n-ary relation extraction with graph lstms. Trans. Associat. Comput. Linguist..

[30] C. Walker, S. Strassel, J. Medero, K. Maeda, Ace 2005 multilingual training corpus, Linguistic Data Consortium, Philadelphia 57 (2006).

[31] L. Song, Y. Zhang, Z. Wang, D. Gildea, N-ary relation extraction using graph-state lstm, in: Proceedings of the 2018 Conference on Empirical Methods in Natural Language Processing, Association for Computational Linguistics, 2018, pp. 2226–2235.

[32] Theano Development Team, Theano: A Python framework for fast computation of mathematical expressions, arXiv e-prints abs/1605.02688 (2016).

[33] Kingma D.P., Ba J.L. (2014). Adam: amethod for stochastic optimization. ICLR.

[34] Mikolov T., Sutskever I., Chen K., Corrado G.S., Dean J. (2013). Distributed representations of words and phrases and their compositionality. Adv. Neural Inf. Process. Syst..

[35] Chelba C., Mikolov T., Schuster M., Ge Q., Brants T., Koehn P., Robinson T. (2014). One billion word benchmark for measuring progress in statistical language modeling. INTERSPEECH.

[36] S. Tokui, K. Oono, S. Hido, J. Clayton, Chainer: a next-generation open source framework for deep learning, in: Proceedings of Workshop on Machine Learning Systems (LearningSys) in the Twenty-Ninth Annual Conference on Neural Information Processing Systems (NIPS), vol. 5, 2015, pp. 1–6

[37] Noreen E.W. (1989). Computer-intensive Methods for Testing Hypotheses.

[38] S. Vashishth, M. Bhandari, P. Yadav, P. Rai, C. Bhattacharyya, P. Talukdar, Incorporating syntactic and semantic information in word embeddings using graph convolutional networks, in: Proceedings of the 57th Conference of the Association for Computational Linguistics, Association for Computational Linguistics, Florence, Italy, 2019, pp. 3308–3318.

[39] I. Iacobacci, M.T. Pilehvar, R. Navigli, SensEmbed: Learning sense embeddings for word and relational similarity, in: Proceedings of the 53rd Annual Meeting of the Association for Computational Linguistics and the 7th International Joint Conference on Natural Language Processing (Volume 1: Long Papers), Association for Computational Linguistics, Beijing, China, 2015, pp. 95–105. URL https://www.aclweb.org/anthology/P15-1010. doi:10.3115/v1/P15-1010.

[40] J. Li, D. Jurafsky, Do multi-sense embeddings improve natural language understanding?, in: Proceedings of the 2015 Conference on Empirical Methods in Natural Language Processing, Association for Computational Linguistics, Lisbon, Portugal, 2015, pp. 1722–1732. URL https://www.aclweb.org/anthology/D15-1200. doi:10.18653/v1/D15-1200.

[41] L. Qiu, K. Tu, Y. Yu, Context-dependent sense embedding, in: Proceedings of the 2016 Conference on Empirical Methods in Natural Language Processing, Association for Computational Linguistics, Austin, Texas, 2016, pp. 183–191. URL https://www.aclweb.org/anthology/D16-1018. doi:10.18653/v1/D16-1018.

[42] R. Socher, B. Huval, C.D. Manning, A.Y. Ng, Semantic compositionality through recursive matrix-vector spaces, in: Proceedings of the 2012 Joint Conference on Empirical Methods in Natural Language Processing and Computational Natural Language Learning, Association for Computational Linguistics, Jeju Island, Korea, 2012, pp. 1201–1211. https://www.aclweb.org/anthology/D12-1110.

[43] K.M. Hermann, P. Blunsom, The role of syntax in vector space models of compositional semantics, in: Proceedings of the 51st Annual Meeting of the Association for Computational Linguistics (Volume 1: Long Papers), Association for Computational Linguistics, Sofia, Bulgaria, 2013, pp. 894–904. URL https://www.aclweb.org/anthology/P13-1088.

[44] R. Socher, A. Karpathy, Q.V. Le, C.D. Manning, A.Y. Ng, Grounded compositional semantics for finding and describing images with sentences, Trans. Associat. Comput. Linguist. 2 (2014) 207–218. URL https://www.aclweb.org/anthology/Q14-1017. doi:10.1162/tacl_a_00177.

[45] A. Eriguchi, K. Hashimoto, Y. Tsuruoka, Tree-to-sequence attentional neural machine translation, in: Proceedings of the 54th Annual Meeting of the Association for Computational Linguistics (Volume 1: Long Papers), Association for Computational Linguistics, Berlin, Germany, 2016, pp. 823–833. URL https://www.aclweb.org/anthology/P16-1078. doi:10.18653/v1/P16-1078.

[46] K.S. Tai, R. Socher, C.D. Manning, Improved semantic representations from tree-structured long short-term memory networks, in: Proceedings of the 53rd Annual Meeting of the Association for Computational Linguistics and the 7th International Joint Conference on Natural Language Processing (Volume 1: Long Papers), Association for Computational Linguistics, Beijing, China, 2015, pp. 1556–1566. URL https://www.aclweb.org/anthology/P15-1150. doi:10.3115/v1/P15-1150.

[47] E. Strubell, P. Verga, D. Andor, D. Weiss, A. McCallum, Linguistically-informed self-attention for semantic role labeling, in: Proceedings of the 2018 Conference on Empirical Methods in Natural Language Processing, Association for Computational Linguistics, Brussels, Belgium, 2018, pp. 5027–5038. URL https://www.aclweb.org/anthology/D18-1548. doi:10.18653/v1/D18-1548.

[48] M. Bansal, K. Gimpel, K. Livescu, Tailoring continuous word representations for dependency parsing, in: Proceedings of the 52nd Annual Meeting of the Association for Computational Linguistics (Volume 2: Short Papers), Association for Computational Linguistics, Baltimore, Maryland, 2014, pp. 809–815. URL https://www.aclweb.org/anthology/P14-2131. doi:10.3115/v1/P14-2131.

[49] M. Faruqui, J. Dodge, S.K. Jauhar, C. Dyer, E. Hovy, N.A. Smith, Retrofitting word vectors to semantic lexicons, in: Proceedings of the 2015 Conference of the North American Chapter of the Association for Computational Linguistics: Human Language Technologies, Association for Computational Linguistics, Denver, Colorado, 2015, pp. 1606–1615. URL https://www.aclweb.org/anthology/N15-1184. doi:10.3115/v1/N15-1184.

[50] I. Vulić, Injecting lexical contrast into word vectors by guiding vector space specialisation, in: Proceedings of The Third Workshop on Representation Learning for NLP, Association for Computational Linguistics, Melbourne, Australia, 2018, pp. 137–143. https://www.aclweb.org/anthology/W18-3018. doi:10.18653/v1/W18-3018.

[51] I. Vulić, G. Glavaš, N. Mrkšić, A. Korhonen, Post-specialisation: Retrofitting vectors of words unseen in lexical resources, in: Proceedings of the 2018 Conference of the North American Chapter of the Association for Computational Linguistics: Human Language Technologies, Volume 1 (Long Papers), Association for Computational Linguistics, New Orleans, Louisiana, 2018, pp. 516–527. URL https://www.aclweb.org/anthology/N18-1048. doi:10.18653/v1/N18-1048.

[52] Kipf T.N., Welling M. (2017). Semi-supervised classification with graph convolutional networks. ICLR.

[53] M. Yasunaga, R. Zhang, K. Meelu, A. Pareek, K. Srinivasan, D. Radev, Graph-based neural multi-document summarization, in: Proceedings of the 21st Conference on Computational Natural Language Learning (CoNLL 2017), Association for Computational Linguistics, Vancouver, Canada, 2017, pp. 452–462. URL https://www.aclweb.org/anthology/K17-1045. doi:10.18653/v1/K17-1045.

[54] Y. Zhang, P. Qi, C.D. Manning, Graph convolution over pruned dependency trees improves relation extraction, in: Proceedings of the 2018 Conference on Empirical Methods in Natural Language Processing, Association for Computational Linguistics, Brussels, Belgium, 2018, pp. 2205–2215. URL https://www.aclweb.org/anthology/D18-1244. doi:10.18653/v1/D18-1244.

[55] E. Voita, D. Talbot, F. Moiseev, R. Sennrich, I. Titov, Analyzing multi-head self-attention: Specialized heads do the heavy lifting, the rest can be pruned, in: Proceedings of the 57th Annual Meeting of the Association for Computational Linguistics, Association for Computational Linguistics, Florence, Italy, 2019, pp. 5797–5808. URL https://www.aclweb.org/anthology/P19-1580. doi:10.18653/v1/P19-1580.

